# How to check a simulation study

**DOI:** 10.1093/ije/dyad134

**Published:** 2023-10-13

**Authors:** Ian R White, Tra My Pham, Matteo Quartagno, Tim P Morris

**Affiliations:** MRC Clinical Trials Unit at UCL, London, UK; MRC Clinical Trials Unit at UCL, London, UK; MRC Clinical Trials Unit at UCL, London, UK; MRC Clinical Trials Unit at UCL, London, UK

**Keywords:** Simulation studies, Monte Carlo, graphics for simulation, avoiding errors

## Abstract

Simulation studies are powerful tools in epidemiology and biostatistics, but they can be hard to conduct successfully. Sometimes unexpected results are obtained. We offer advice on how to check a simulation study when this occurs, and how to design and conduct the study to give results that are easier to check. Simulation studies should be designed to include some settings in which answers are already known. They should be coded in stages, with data-generating mechanisms checked before simulated data are analysed. Results should be explored carefully, with scatterplots of standard error estimates against point estimates surprisingly powerful tools. Failed estimation and outlying estimates should be identified and dealt with by changing data-generating mechanisms or coding realistic hybrid analysis procedures. Finally, we give a series of ideas that have been useful to us in the past for checking unexpected results. Following our advice may help to prevent errors and to improve the quality of published simulation studies.

Key Messages

- Some standard techniques can help to avoid or to correct errors in simulation studies.
- Simulation studies should be designed to include well-understood settings in which results can be checked against known answers.
- Simulation studies should be coded carefully and simulated data sets should be checked.
- Estimates from simulation studies should be checked carefully for outliers and failed estimation.
- We suggest various ways to check surprising results.

## Introduction

Simulation studies are widely used to evaluate statistical methods in epidemiology and biostatistics. We, like many epidemiologists and biostatisticians, often find ourselves not believing, or being sceptical of, the results of a simulation study done by a colleague, a student or ourselves. This article makes a number of suggestions based on our experience of discovering mistakes in our own and collaborators’ simulation studies. Our aims are to provide a structured approach to determining whether the results really are wrong and, if so, to identifying the source of the errors and how they can be corrected.

We can divide the execution of a simulation study into three stages: ‘design’ (identifying the aims, data-generating mechanisms, estimands, methods of analysis and performance measures^1^); ‘conduct’ (writing the code to simulate multiple data sets and analyse each one, yielding a data set of estimates); and ‘analysis’ (computing the performance measures from the estimates data set). Our advice is intended to apply after the analysis stage is complete. Often the most useful advice is to go back and modify the design or conduct of the simulation study. We therefore structure our advice into these three stages of the simulation study. The advice given under ‘design’ and ‘conduct’ is equally applicable at the start of a simulation study. Some parts of our approach are very widely applicable whereas other parts are tailored to a specific setting: they are not all appropriate in every setting.

In this article, we first set out some established terminology for simulation studies and describe a running example simulation study. We then give our points of advice, arranged as ‘design’, ‘conduct’ and ‘analysis’. Most of the points of advice are illustrated using the running example. Readers are encouraged to read the illustrations alongside the Stata and/or R code and output available in the Supplementary Materials (available as Supplementary data at *IJE* online) and at https://github.com/UCL/simcheck. We end with a short summary.

## Terminology

Table 1 summarizes various terms used in simulation studies, including the aspects of a simulation study, the data sets used and some performance measures.^1^

**Table 1.** Terms used in simulation studies^1^

Term	Explanation
**Aspects of a simulation study** ^1^	
Aims	What question(s) the simulation study addresses
Data-generating mechanisms	How the simulated data sets are to be generated
Estimands	The quantity or quantities to be estimated by the analysis of each simulated data set
Methods of analysis	How the simulated data sets are to be analysed: typically producing a point estimate, its standard error estimate and a CI
Performance measures	How the performance of the methods of analysis is to be summarized
Implementation	How the simulation is to be performed, including the software used, the number of repetitions and the handling of random number states
**Data sets involved in a simulation study** ^1^	
Simulated data set	A data set produced by one of the data-generating mechanisms in one repetition
Estimates data set	A data set containing results of each method of analysis for each simulated data set across many repetitions, used to estimate performance
States data set	A data set containing random number states for each simulated data set, that can be used to recreate any simulated data set
Performance measures data set	A data set containing the estimated performance measures for each data-generating mechanism and each method of analysis
**Some performance measures**	
Bias	How the mean point estimate differs from the true estimand value
Empirical standard error	The standard deviation of the point estimates in an estimates data set
Model-based standard error	The average^a^ standard error estimate in an estimates data set
Relative error in model-based standard error	The difference between the model-based standard error and the empirical standard error, expressed as a fraction of the latter
Coverage	The proportion of CIs that include the true estimand value

a Strictly, the root mean square of the standard error estimates.

## Example

**Figure 1.**
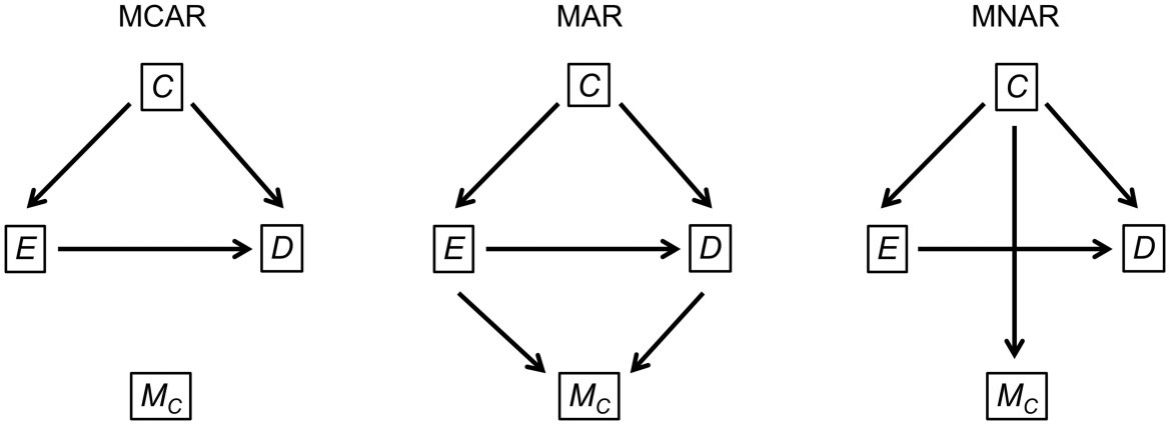
Directed acyclic graphs showing the data-generating mechanisms used in the example simulation study under three missing data mechanisms: missing completely at random (MCAR), missing at random (MAR) and missing not at random (MNAR). C is the confounder, E is the exposure, D is the outcome and M_C_ denotes whether C is missing

We illustrate our advice using a hypothetical simulation study exploring the use of multiple imputation for epidemiological data with missing values in the confounders (Table 2 and Figure 1). The study aims to compare multiple imputation with complete case analysis and is loosely based on a published study.^2^

**Table 2.** Key features of the example simulation study

Aim	To compare multiple imputation with complete case analysis
Data-generating methods^a^	Quantitative confounder C is drawn from a standard Normal distribution. Binary exposure E and binary outcome D are drawn from logistic models depending on C (so E does not cause D). Values of C are made missing, initially using a missing completely at random model. Parameters to be varied are the marginal probabilities of E and D, the strength of the dependence of E and D on C, and the missing data mechanism. The sample size of 500 is fixed.
Estimand	The log odds ratio between E and D, conditional on C. Its true value is zero.
Methods	Logistic regression of D on E and C, using full data before data deletion in C complete cases (excluding cases with missing C) multiply imputed data to handle missing values of C—various imputation models may be used.
Performance measures	Bias, empirical standard error, relative error in model-based standard error, coverage.
Implementation	1000 repetitions—advice on choosing this is available.^1^

a Specific values used in the data-generating mechanisms can be found in the code.

## Design: planning the simulation

### (i) Include a setting with known properties

We frequently do simulation studies to learn the properties of methods of analysis, but often we can identify a particular data-generating mechanism and a particular method of analysis where we know some aspect of the answer. This requires knowledge of the statistical properties of the methods. We can then check our results against these known answers.

#### Example

In the multiple imputation simulation study, we included analysis of the full data before data deletion. Assuming the analysis procedure is correct, we expect this to be unbiased with correct coverage and more precise than any methods of analysis of the incomplete data. We also include a complete case analysis in a scenario with data missing completely at random, which is expected to be unbiased with correct coverage and less precise than multiple imputation methods of analysis.^2^

The above statements about lack of bias are not strictly correct, since logistic regression is biased in small samples. We could therefore include a further setting with a larger sample size.

## Conduct: coding the simulation

### (ii) Write well-structured code

It is helpful to write code that separates data generation, data analysis and computation of performance measures, so that each part can be studied separately. Code should be well commented to help collaborators and the coder’s future self; the final code should later be published and comments will then help the general reader.

#### Example

The Stata script simcheck02.do and the R script simcheck02.R separate out data generation and data analysis. We will add the calculation of performance measures later.

### (iii) Study a single very large data set

The code for data generation should next be used to generate a single very large data set. Viewing descriptive statistics (e.g. histogram of a continuous outcome, cross-tabulation of exposure by outcome event) allows a check that the data match one’s intentions.

In many cases, the model used to generate the data can be directly fitted to the simulated data. This should recover the parameters of the data-generating model such that CIs for the results usually include the known true values.

Then the simulation code should be used to do the analyses. The results should be carefully checked for correct background information (e.g. is the number of observations correct?) and for credibility. Again, where methods are known to be correct, CIs for the results should usually include the known true values.

#### Example

The scripts simcheck03.do and simcheck03.R generate a single data set of size 100 000 using a particular data-generating mechanism. They include standard descriptive statistics such as a cross-tabulation of D against E, showing that there is an unconditional association between D and E (log odds ratio = 0.74 in Stata and 0.70 in R). This is important because one way in which imputation procedures could perform badly is by failing to control for confounding. The scripts also show that the logistic regressions of E and D on C have coefficient values very near to the values in the data-generating mechanism and that the analysis program runs successfully.

### (iv) Run the simulation with a small number of repetitions

The simulation code should next be checked with a small number of repetitions: three repetitions are often enough at this stage. The screen output should be switched on so that it can be studied. The size of the simulated data set should be checked for each repetition.

It is useful to verify that the second and third repetitions produce different data and results. Sometimes simulation code wrongly sets the random number state after starting the first repetition. If (for example) this is at the end of a repetition, then the second and third repetitions would produce identical data and results.

This is the first time that we have created an estimates data set so it is timely to check that the estimates data set has the right structure, is indexed with the correct simulation repetition number and contains values that match the values reported in the screen output. For example, sometimes users store a variance when they meant to store a standard error estimate. Confusion can arise if screen output displays exponentiated parameters (odds ratios) but estimates are stored on the estimation scale (log odds ratios).

#### Example

The scripts simcheck04.do and simcheck04.R run three repetitions of the simulation. The results look sensible and match the screen output.

### (v) Anticipate analysis failures

If a certain method of analysis can be anticipated to cause an error in some simulated data sets (e.g. perfect prediction in a logistic regression), the code should be written to capture the error so that the simulation does not halt. The method failure (with error code) should be stored in the estimates data set. Strategies for handling failures are described in later points.

#### Example

The scripts simcheck05.do and simcheck05.R recognize that either the imputation step or the model fitting to the imputed data may fail. They therefore detect either of these failures and post missing values to the estimates data set.

### (vi) Make it easy to recreate any simulated data set

The estimates data set should include an identifier for the simulated data set alongside every estimate. If we can recreate the simulated data set for any particular identifier, then we can explore method failures and outliers (see points below). There are two ways to do this. One way is to store the random number state at the start of each data generation in a states data set so that the user can recreate any simulated data set. The alternative is to store every simulated data set.

Some analyses, such as multiple imputation, also use random numbers. Recreating such analyses requires resetting the appropriate random number state. One way is to recreate the simulated data set (as above) and then repeat the analysis; if multiple analyses use random numbers then they must all be repeated in the original order. The alternative is to store the random number state at the start of each stochastic analysis.

#### Example

The scripts simcheck06.do and simcheck06.R store the random number states in a separate file. They then show how to reconstruct the third simulated data set.

## Analysis: method failures and outliers

Once the code is written and tested, we are ready to run the simulation study and start looking at the results. We first discuss detecting and handling method failures and outliers.

### (vii) Count and understand method failures

For each data-generating mechanism and method, the user should identify what fraction of repetitions led to failed estimation. The reasons for failed estimation need to be understood and the code should be improved if possible. That is, a bad optimization routine should be improved if it makes a method appear to fail. Unexpected results may be specific to particular software or packages.

#### Example

The scripts simcheck07.do and simcheck07.R use a different data-generating mechanism from previous runs. Inspecting the estimates data set shows four method failures in Stata and two in R. On closer exploration of the simulated data sets, we find that these data are very sparse, having either no exposed individuals or no outcome events in the individuals with observed C (a random positivity violation that may not be intended), and this is causing the complete case analysis to fail. The R function glm behaves differently and returns estimates even in the absence of outcome events.

The sensible conclusion (in our setting in which positivity violations are not of main interest) is that the data-generating mechanism is too extreme and should be changed to generate more outcome events. For the purposes of illustration, we do not do this yet.

### (viii) Look for outliers

It is important to examine the estimates data set carefully. A useful visual device is a scatter plot of the standard error estimate against the point estimate over all repetitions, separated by the data-generating mechanism and method. This scatter plot can identify the presence of outliers. Estimates can be outliers for the point estimate or the model-based standard error (or both). Such outliers are frequent causes, respectively, of unexpected bias and of unexpected error in the model-based standard error. A small number of outliers by themselves do not affect coverage and often researchers are puzzled by, for example, a model-based standard error being apparently very large without any impact on coverage.

#### Example

**Figure 2.**
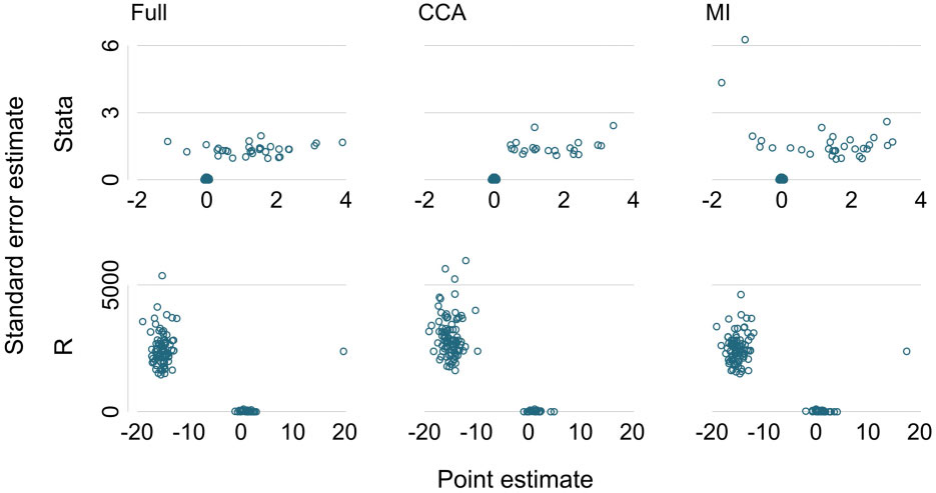
Looking for outliers in the estimates data from simcheck08.do and simcheck08.R. CCA, complete case analysis; MI, multiple imputation

The scripts simcheck08.do and simcheck08.R explore the estimates data sets produced by simcheck07.do and simcheck07.R. They plot the standard error estimates against the point estimates by method of analysis. Results differ between the packages. In Stata (Figure 2, upper part), a substantial number of data sets have standard error estimates equal to zero, which indicates a problem with the analysis. Further inspection shows these data sets also have estimated coefficients equal to zero. In R (Figure 2, lower part), a substantial number of data sets have very large standard error estimate (2000–5000). These also have large point estimates, mostly between –10 and –20, some near +20.

We could change these outlying standard error estimates and their associated point estimates to missing values, but it is more important to understand their cause. We do this next.

### (ix) Understand outliers

If outliers are found, it is helpful to open or recreate one or more of the corresponding simulated data sets. The user should verify the outlying estimate and explore the reasons for it, such as by checking details of the analysis output and by supplementary analysis such as exploring model residuals or imputed data values.

#### Example

Scripts simcheck09.do and simcheck09.R each pull out one particular problem data set identified in point (viii). In both cases, the problem data set has no events among individuals with observed confounders. The different outputs from the two software packages (Figure 2) arise from the packages’ different handling of this problem. Stata has detected perfect prediction,^3^ has dropped the exposure variable E from the model and has reported zero values for the point estimate and its standard error estimate. R has performed estimation regardless, has found the parameter estimate going towards plus or minus infinity without achieving convergence and has reported values when approximate convergence is achieved.

Solutions to these problems are discussed next.

### (x) Deal with outliers

Any outliers are likely to strongly affect estimates of performance. It may be appropriate to change the data-generating mechanism to avoid outlying estimates. Otherwise, if outlying estimates would not be believed or reported in practice—that is, if the issue would be detected and the method of analysis not used—then they should not be included in the analysis of the simulation results. One way to do this is to exclude simulated data sets that result in outlying estimates. However, this can introduce a selection bias because the excluded simulated data sets are unlikely to be representative. An alternative is to code an automatic ‘backup procedure’ when a method returns an absurd result. This changes the method being investigated from a pure method to a hybrid procedure and it should make performance measures more relevant to practice. Alongside any of these approaches, the number of method failures or outliers is a useful additional performance measure.

#### Example

One way to avoid the problems seen in the multiple imputation simulation study is to increase the proportion of exposed away from the sparse case seen. We adopt this approach in the remaining points.

Alternatively, if we were interested in the sparse case, we should ask whether outlying estimates might make sense in practice. An analyst might accept a log odds ratio of –15 and report an estimated odds ratio of zero, which implies either no events in the exposed group or no non-events in the unexposed. However, they should certainly not accept the very wide 95% CI. Instead they would probably use exact methods^4^ to generate a more correct CI. We could therefore code such exact methods into our simulation study as a backup procedure if extreme estimates are found.

A different way to fix the analysis, and a more convenient solution for the simulation study, is to handle perfect prediction by using penalized logistic regression.^5^ This could be done as a backup procedure in analyses exhibiting a problem or in all analyses. The latter is illustrated in simcheck10.do and simcheck10.R.

## Analysis: unexpected findings

### (xi) Check Monte Carlo errors

Sometimes, some simulation findings are hard to believe: for example, a method selected to be unbiased appears to be biased or one method appears to be more precise than another when it should be less precise. In this case, it is important to look at the Monte Carlo errors and decide whether the findings are compatible with Monte Carlo error.

#### Example

The scripts simcheck11.do and simcheck11.R are our first complete runs of a simulation study avoiding sparse data. Results for bias suggest a larger bias in complete case analysis (e.g. in Stata –0.094) than in the other methods (–0.068 full data, +0.063 multiple imputation). Given that we are simulating under missing completely at random, we expect complete case analysis to be unbiased. Instead of concluding that our code is wrong, we should spot that these results are compatible with Monte Carlo error—that is, the observed bias for complete case analysis is <2 Monte Carlo standard errors (0.049 × 2 = 0.098) and hence is perfectly compatible with zero bias. In fact these results were produced with just 100 repetitions. To detect a bias of this magnitude, more repetitions (say 1000) are needed.

### (xii) Why are model-based standard errors wrong?

If model-based standard errors disagree with the empirical standard errors, it is worth considering whether the sources of variation in the data-generating mechanism and analysis correspond. For example, in a missing data simulation study, if each repetition under a given data-generating mechanism starts from the same full data set, then uncertainty due to the full data set will be reflected in the model-based standard error but not in the empirical standard error, making them not comparable regardless of the analysis used.

#### Example

The scripts simcheck12.do and simcheck12.R demonstrate this issue. Previous scripts drew a new full data set for each repetition, but these scripts create each simulated data set by deleting values from the same full data set. The model-based standard errors (e.g. in Stata 0.52 and 0.46 for complete case analysis and multiple imputation, respectively) are found to be substantially larger than the empirical standard errors (0.27 and 0.17). This is because the model-based standard errors account for sampling variation in the full data set whereas the sampling variation in the full data set does not exist in the simulation study so is not reflected in the empirical standard error. One solution here is to generate a new full data set for each repetition.

### (xiii) Why is coverage poor?

If coverage is poor, it is helpful to identify whether it is driven by bias, by intervals of the wrong width or both. Zip plots are a useful visualization devices for this purpose. They plot each interval, ordered according to compatibility with the true value, giving the impression of a ‘zip’ (or ‘zipper’).^1^

#### Example

**Figure 3.**
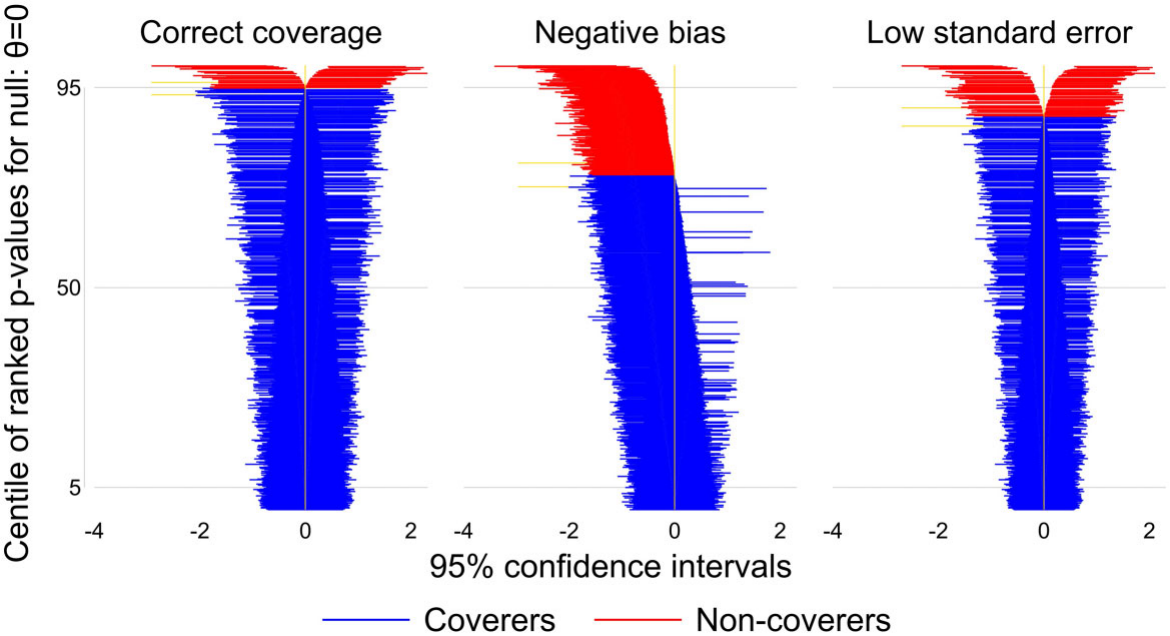
Zip plots illustrating correct coverage, undercoverage due to bias and undercoverage due to low standard error

Figure 3 shows illustrative zip plots, created by Stata script simzipplot.do and using the new siman suite in Stata (available at https://github.com/UCL/siman); a similar plot can be achieved using the rsimsum package in R. One thousand CIs are ranked by their *P*-values and coloured red if they fail to cover the true value: zero. The left-hand panel shows correct coverage. The middle panel shows low coverage due to negative bias, with most CIs (including an excess of non-covering ones) lying to the left of the true value. The right-hand panel shows low coverage due to low standard error, with an excess of non-covering CIs occurring symmetrically.

### (xiv) Why are power and type I error wrong?

Somewhat different errors can arise when focusing on test characteristics. Some pitfalls are to interchange power and type I error, or to mistake one-sided and two-sided type I tests.

#### Example

Errors are easy to make if simulating a non-inferiority trial.^6^ Here, generating data under no treatment effect gives the power whereas generating data with a treatment effect equal to the margin gives the type I error rate.

### (xv) When do unexpected findings occur?

If some findings remain hard to believe, it may be helpful to ask under what settings these findings occur. For example, if they occur only when the data-generating mechanism includes a particular source of variation, then maybe analyses are not allowing for this source of variation.

#### Example

We encountered this problem in a simulation study in a meta-analysis. Roughly, data were generated under a common-effect model and a random-effects model.^7^ However, model-based standard errors were larger than empirical standard errors when data were generated under the random-effects model. It turned out that the random effects were being generated once at the start of the simulation study and not changed thereafter—effectively a fixed-effect rather than random-effects model. As a result, the empirical standard error did not account for variation in the random effects whereas the model-based standard errors did. Generating the random effects afresh at each repetition fixed the problem.

### (xvi) General checking method

If after the above steps the results of the simulation study are still in doubt, it can be useful to recode the simulation study in a different statistical package or have a different person code it. Sometimes another closely related simulation study that has been published with code is helpful. The user should first check that the published code does reproduce the published results. They can then change the published data-generating mechanisms to match those in the current simulation study (as closely as possible), run them and compare the results. Alternatively they can change their own data-generating mechanism parameters to match the published ones (as closely as possible) and run them and compare the results. This should help to narrow down where any errors are occurring. However, if code has been carefully checked and still gives an unexpected result, it is important to consider that the findings may be genuine showing that the theory may be wrong.

## A successful simulation study

The scripts simcheck99.do and simcheck99.R show a successful simulation study using three missing data mechanisms: MCAR, missing completely at random; MAR, missing at random; and MNAR, missing not at random. There are many MAR and MNAR mechanisms, and our results apply only to the particular mechanisms chosen. Results from the Stata code are shown in Table 3. Entries in italics show results to be compared against existing knowledge: for example, multiple imputation has empirical standard error between full data and complete case analysis. Entries in bold show the key findings, namely (i) complete case analysis shows evidence of bias under MAR only; (ii) multiple imputation (MI) shows evidence of bias under MNAR only; and (iii) coverage of 95% CIs is acceptable for both methods, despite these biases and small errors in the model-based standard error.

**Table 3.** Results of final simulation study

Performance measure	Data-generating mechanism	Full data	Complete case analysis	Multiple imputation
Bias in point estimate (Monte Carlo error < 0.02)	MCAR	*0.00*	*–0.01*	**0.00**
	MAR	*–0.02*	**–0.16**	**–0.02**
	MNAR	*–0.01*	**–0.03**	**0.05**
Empirical standard error (Monte Carlo error < 0.02)	MCAR	*0.43*	*0.52*	*0.45*
	MAR	*0.43*	*0.58*	*0.46*
	MNAR	*0.43*	*0.54*	*0.46*
Relative error in model-based standard error (Monte Carlo error = 2%)	MCAR	*–4%*	*–4%*	–4%
	MAR	*–5%*	**–6%**	–3%
	MNAR	*–5%*	–3%	–6%
Coverage of 95% CI (Monte Carlo error < 1%)	MCAR	*95%*	*95%*	95%
	MAR	*94%*	**95%**	**94%**
	MNAR	*94%*	**96%**	**93%**

MAR, missing at random; MCAR, missing completely at random; MNAR, missing not at random. Entries in italics show results to be compared against existing knowledge; entries in bold show the key findings.

## Conclusion

Our advice covers a number of issues that have caused difficulties in simulation studies that we have worked on, and we hope they are useful to others. Other useful literature on simulation studies is available.^1^^,^^8^ Our motivation was to explain surprising simulation results after a simulation study is run, but quality should be designed in from the start. We stress that simulation studies should be designed to be easy to check and should be checked repeatedly during the conduct and analysis stages.

## Ethics approval

Ethics approval is not needed for this study since it uses no real data.

## Supplementary Material

dyad134_Supplementary_Data

## Data Availability

No patient data were generated or analysed in support of this research.

## References

[1] Morris TP , White IR, Crowther MJ. Using simulation studies to evaluate statistical methods. Stat Med 2019;38:2074–102. 30652356 10.1002/sim.8086 PMC6492164

[2] White IR , Carlin JB. Bias and efficiency of multiple imputation compared with complete-case analysis for missing covariate values. Stat Med 2010;29:2920–31. 20842622 10.1002/sim.3944

[3] Heinze G , Schemper M. A solution to the problem of separation in logistic regression. Stat Med 2002;21:2409–19. 12210625 10.1002/sim.1047

[4] Mehta CR , Patel NR. Exact logistic regression: Theory and examples. Stat Med 1995;14:2143–60. 8552893 10.1002/sim.4780141908

[5] Firth D. Bias Reduction of Maximum Likelihood Estimates. Biometrika 1993;80:27–38.

[6] Schumi J , Wittes JT. Through the looking glass: understanding non-inferiority. Trials 2011;12:106. 21539749 10.1186/1745-6215-12-106 PMC3113981

[7] Higgins JPT , Thompson SG, Spiegelhalter DJ. A re-evaluation of random-effects meta-analysis. J R Stat Soc Ser A Stat Soc 2009;172:137–59. 10.1111/j.1467-985X.2008.00552.x PMC2667312 19381330

[8] Mayer C , Perevozskaya I, Leonov S et al Simulation practices for adaptive trial designs in drug and device development. Stat Biopharm Res 2019;11:325–35.

